# Respiratory Muscle Strength and Exercise Performance in Cystic Fibrosis–A Cross Sectional Study

**DOI:** 10.3389/fped.2018.00244

**Published:** 2018-09-04

**Authors:** Aleksandar Sovtic, Predrag Minic, Gordana Markovic-Sovtic, Goran Z. Trajkovic

**Affiliations:** ^1^Department of Pulmonology, Mother and Child Health Institute of Serbia, Belgrade, Serbia; ^2^School of Medicine, University of Belgrade, Belgrade, Serbia; ^3^Institute for Medical Statistics, Belgrade, Serbia

**Keywords:** cystic fibrosis, exercise testing, spirometry, whole body plethysmography, respiratory muscle strength

## Abstract

**Introduction:** Decreased respiratory muscle strength in patients with cystic fibrosis (CF) may cause progressive exercise intolerance during cardiopulmonary exercise testing (CPET), and may contribute to the development of chronic respiratory insufficiency. The aim of this study is to evaluate exercise tolerance during CPET of children and adults with clinically stable CF who exhibit different respiratory muscle strength.

**Methods:** Sixty-nine clinically stable CF subjects aged 8–33 years underwent spirometry, body plethysmography, CPET, and respiratory muscle strength measurement. Respiratory muscle strength was measured using maximal inspiratory pressures (Pi_max_) and maximal expiratory pressures (Pe_max_). Participants were stratified into three groups according to Pi_max_ values:below normal (≤80% predicted), normal (81–100% predicted), and above normal (>100% predicted). A similar stratification of participants was made according to Pe_max_ values. The oxygen consumption on peak load (VO_2peak_) was expressed relative to BM (VO_2peak_/kg), relative to BM raised by the exponent of 0.67 (VO_2peak_/kg^0.67^) and as log-linear adjustment of VO_2peak_ (VO_2peak/kg−alo_).

**Results:** Participants with low Pe_max_ values had a lower mean maximum load per kilogram/predicted (W_max_; *p* = 0.001) VO_2peak_/kg (*p* = 0.006), VO_2peak_/kg^0.67^ (*p* = 0.038) and VO_2peak/kg−alo_ (*p* = 0.001). There were no significant differences in exercise tolerance parameters with regard to Pi_max_ values. Stepwise multiple linear regressions confirmed that Pe_max_ (*B* = 24.88, β = 0.48, *p* < 0.001) was the most powerful predictor of W_max_. There were no statistically significant differences in age, lung function parameters, exacerbation score, or respiratory muscle strength according to gender.

**Conclusions:** In subjects with clinically stable CF, expiratory muscle strength is associated with a decrease in exercise performance during CPET and can predict exercise intolerance. Increase in expiratory muscle strength by patient specific rehabilitation protocols would result in improvement of exercise tolerance.

## Introduction

Lung hyperinflation, decreased lung function, and malnutrition are leading causes of progressive exercise intolerance in patients with cystic fibrosis (CF) (1). Respiratory muscle weakness may also contribute to the development of chronic respiratory insufficiency (2). However, previous studies have not successfully confirmed a significant relationship between respiratory muscle strength, nutritional status, lung function, and exercise tolerance (3).

Measurement of maximal respiratory pressures serves to determine whether respiratory muscle weakness exists and to quantify its severity. It is cheap and simple way to evaluate respiratory muscle force. The results of studies published to date regarding muscle strength in CF patients are contradictory (2–5). Some studies evaluating maximum static respiratory pressures have indicated preserved respiratory muscle strength in CF patients, although there are opposing opinions (2, 3, 6, 7). The preserved strength was attributed to respiratory muscles being exercised through chronic coughing and an increased ventilatory load. Opposite findings were presumably not related to metabolic reasons or lower serum androgen concentrations (8, 9). Patients with mild CF exhibit significantly diminished maximal inspiratory pressure (Pi_max_) and maximal expiratory pressure (Pe_max_) compared with healthy controls (7). The relationship between respiratory muscle strength and the results of modified shuttle tests has already been investigated (10), but the relationship between respiratory muscle strength and the results of cardiopulmonary exercise testing (CPET) had not been investigated until now.

The aim of this study is to evaluate exercise tolerance during CPET of children and adults with clinically stable CF who exhibit different respiratory muscle strength. We hypothesized that the patients with more severe lung disease would have decreased respiratory muscle strength, which may have a negative influence on exercise tolerance.

## Methods

### Study design

This was a cross sectional study of respiratory muscle strength and exercise tolerance, conducted from August 2016 to August 2017 in Mother and Child Health Institute of Serbia, the national CF center. Patients and their legal guardians signed informed consent documents before participating in the study. The protocol was approved by the Ethics Committee of the Mother and Child Health Institute of Serbia (number 82/16). All subjects and their legal guardians gave written informed consent in accordance with the Declaration of Helsinki.

### Subjects

For this study, 71 subjects with mild to moderate CF lung disease aged 8–33 years were screened. Although this represents a relatively inhomogeneous age group, all subjects had previous experience with measurements as defined by the protocol of the study. All participants were clinically stable, without symptoms of pulmonary exacerbation, defined as systemic antibiotic use for at least 6 weeks prior to the start of the study. Although previous studies had not shown that pulmonary exacerbation has a negative impact on muscle strength, these studies showed it has a negative influence on lung function and exercise tolerance (11–13). Height and body mass (BM) were recorded. Adiposity was expressed as Z-score of body mass index (BMI). Body mass was also raised to the 0.67.

Measurements of lung function and respiratory muscle strength were taken, and exercise testing was performed in consecutive order, at the same time of the day on each occasion, after a half-hour pause between each procedure. Treatment with bronchodilators was allowed as routine therapy, as well as a regular course of physiotherapy at home prior to the study.

### Lung function and cardiopulmonary exercise testing

Spirometry and whole-body plethysmography were performed on a pneumotach system using a volume-constant method (MasterLab, Jaeger, Würzburg, Germany). The reference equations used for pulmonary function testing were those of Zapletal et al. (14) Participants performed progressive CPET on an electrically braked cycle ergometer (MasterScreen CPX; Jaeger, Würzburg, Germany), following the modified Godfrey protocol to maximum effort. Work increments were planned individually to reach the maximal exercise level by approximately 8–10 min. The determination of maximal effort was based on objective criteria: peak heart rate (HR) >95% HR predicted (210 – age) or respiratory exchange ratio (RER) >1.1. Two patients who did not achieve at least one of these criteria were excluded from the study. Patients breathed through a tightly sealed mask with electronically compensated dead space. Mask was connected to a TripleV sensor. Expired gas passed to an attached metabolic cart (Oxycon pro, Carefusion) with oxygen and carbon dioxide analyzers. Oxygen saturation (SaO_2_) was measured continuously with sensors placed on participants' fingertips (Model 3011; Nonin Medical, *Minneapolis MN*, USA). A computer calculated breath-by-breath tidal volume, respiratory rate and minute ventilation, oxygen consumption (VO_2_), carbon dioxide production (VCO_2_), and RER as well. The oxygen consumption on peak load (VO_2peak_) was expressed in absolute values (L·min^−1^), relative to BM (VO_2peak_/kg) and relative to BM raised by the exponent of 0.67 (VO_2peak_/kg^0.67^). In addition, log-linear adjustment of VO_2peak_ (VO_2peak/kg−alo_) was done as it was proposed by Welsman et al. (15). Ventilation relative to VO_2_ and VCO_2_ was expressed as ventilatory equivalents for O_2_ and CO_2_ (V_E_/VO_2_ and V_E_/VCO_2_). The reference values of peak exercise capacity (W_max_) were those of Wasserman et al. (16) The anaerobic threshold (AT) was determined using the V-slope method (17). Participants were asked to score their sense of breathlessness at W_max_ using a ten-point Borg scale immediately after exercise. Study participants were familiar with CPET, performed on regular annual check-ups, according to Statement of European Cystic Fibrosis Exercise Working Group (18).

### Respiratory muscles strength measurement

Respiratory muscle strength was measured using a handheld mouth pressure meter (MicroRPM, CareFusion Ltd., San Diego, CA, USA) connected to a computer (Puma® software). All procedures were performed with the subjects seated comfortably, and maximum respiratory pressures were expressed as Pi_max_ (maximal inspiratory pressures) and Pe_max_ (maximal expiratory pressures) (19). A maximal static expiratory maneuver was measured from total lung capacity (TLC), and a maximal static inspiratory maneuver from residual volume (RV). Pressure was maintained for at least 1.5 s so that the maximum pressure sustained for 1 s could be calculated. Participants were encouraged to give their best effort during the procedure. Five or more measurements, separated by 2 min of rest, were taken until two reproducible maximal values were obtained. The results were expressed as percentage predicted for age and gender using reference values of Wilson et al. (20).

In order to evaluate differences in lung function and exercise tolerance, participants were stratified into three groups based on Pi_max_ values: group 1 (≤80% predicted), group 2 (81 to 100% predicted), and group 3 (>100% predicted). A similar 3-group stratification was made based on Pe_max_ values.

### Statistical analysis

Statistical analyses were performed at the Institute for Medical Statistics, School of Medicine, Belgrade. Differences between groups were analyzed using one-way analysis of variance (ANOVA). Student's *t*-test was used to examine differences between males and females. Simple linear regression was used to predict W_max_ as the dependent variable. Multiple and stepwise linear regressions were used when several predictors that might explain the model of one dependent variable were present. *P*-values < 0.05 indicated statistical significance. Sample size estimation was performed according to data obtained after evaluation of the results from the first 15 subjects. The power of the one-way ANOVA procedure was 0.920. Data were analyzed using SPSS 21 for Windows and expressed as mean ± SD.

## Results

Data collected from 69 participants with CF (36 males, 33 females) were included in this analysis. The mean participant age was 16.8 ± 6.5 years (range, 8–33 years). Mean values of Pi_max_ and Pe_max_ were within the normal range.

Children and adolescents had significantly higher FEV_1_ (*p* < 0.001), VO_2peak_/kg (*p* = 0.001) VO_2peak/kg−alo_ (*p* < 0.001), than adults. There were no significant differences with regard to participant's age comparing W_max_, VO_2peak/_kg^0.67^, Pi_max_, and Pe_max_ (Table 1).

**Table 1:** Demographic data.

**Mean ± SD**	**Total**	**Male**	**Female**	**Children**	**Adults**
Age (years)	16.8 ± 6.5	17.7 ± 6.5	15.8 ± 6.5	12.3 ± 3.1	23.4 ± 4.1^†^
Exacerbation score (1/year)	1.6 ± 0.7	1.4 ± 0.7	1.7 ± 0.7	1.5 ± 0.7	1.5 ± 0.7
BMI	18.4 ± 3.3	19.2 ± 3.4	17.6 ± 2.9^*^	17.3 ± 3.3	20.3 ± 2.5^†^
FEV_1_ (%)	76.5 ± 27.1	76.7 ± 28.3	76.4 ± 26.2	84.3 ± 25.8	65.1 ± 25.1^†^
FVC (%)	83.0 ± 23.3	83.8 ± 24.9	82.2 ± 21.8	86.5 ± 23.4	77.8 ± 22.6
RV/TLC (%)	167.8 ± 53.3	166.3 ± 57.8	169.4 ± 48.7	158.1 ± 48.4	182, 0 ± 57.7^†^
VO_2peak_/kg	33.2 ± 7.5	35.1 ± 7.8	31.2 ± 6.6^*^	35.6 ± 6.7	29.8 ± 7, 3^†^
VO_2peak_/kg^−0.67^	115.3 ± 28.8	127.2 ± 27.5	102.2 ± 24.4^†^	116.2 ± 27.3	113.9 ± 31.3
VO_2peak_/kg_alo_	4.2 ± 0.3	4.3 ± 0.3	4.1 ± 0.3^†^	4.4 ± 0.3	4.2 ± 0.2^†^
W_max_ (%)	85.1 ± 17.6	83.2 ± 19.4	87.2 ± 15.5	82.7 ± 16.1	88.5 ± 19.5
Pi_max_ (%)	108.1 ± 34.6	102.1 ± 39.3	114.6 ± 27.7	112.1 ± 37.0	102.1 ± 30.3
Pe_max_ (%)	104.3 ± 34.4	100.2 ± 33.6	108.8 ± 35.3	102.9 ± 32.9	106.4 ± 37.1
VE/VCO_2_ W_max_	32.4 ± 4.2	31.4 ± 4.3	33.5 ± 3.7^*^	32.8 ± 3.8	31.9 ± 4.6
BRI W_max_	0.8 ± 0.3	0.9 ± 0.3	0.8 ± 0.2	0.8 ± 0.2	0.9 ± 0.3
SpO_2_ rest (%)	96.7 ± 2.0	96.9 ± 1.8	96.5 ± 2.2	96.9 ± 1.9	96.3 ± 2.2
SpO_2_ W_max_ (%)	91.7 ± 5.4	91.7 ± 4.9	91.7 ± 5.9	93.1 ± 4.4	89.7 ± 6.1^†^

RV/TLC, residual volume/total lung capacity; W_max_, maximal load; VO_2_peak/kg, maximal oxygen consumption/kg; BRI, breathing reserve index; Pi_max_, maximal inspiratory pressure; Pe_max_, maximal expiratory pressure; V_E_/VCO_2_, ventilatory equivalent for carbon dioxide.

* p < 0.05,

† *p < 0.01*.

Participants with decreased Pe_max_ had significantly lower lung function values: lower mean FEV_1_ (*p* < 0.001), and higher mean RV/TLC (*p* = 0.002). Differences were not proved to be statistically significant between Pi_max_ groups.

CPET measurements showed that subjects with low Pe_max_ had lower mean W_max_ (*p* = 0.001), VO_2peak_/kg (*p* = 0.006), VO_2peak_/kg^0.67^ (*p* = 0.038) and VO_2peak−alo_ (*p* < 0.001) values. There were no significant differences in exercise tolerance with regard to Pi_max_. There were no significant differences between groups with regard to oxygen saturation rate SpO_2_ at W_max_ or dyspnea score (Table 2).

**Table 2:** Exercise testing and respiratory muscle strength.

		***N***	**W_***max***_ (%)**	**VO_**2peak**_ml/kg**	**VO_**2peak**_ml/kg^**0.67**^**	**VO_**2peak**_ml/kg-alo**	**Dyspnea score**
Pe_max_ (% pred)	≤80%	15	72.7 ± 17.2	27.9 ± 6.5	98.9 ± 36.6	4.1 ± 0.4	7.3 ± 1.9
	81–100%	22	83.1 ± 16.5	34.6 ± 6.9	121.8 ± 25.7	4.3 ± 0.3	6.6 ± 1.5
	>100	32	92.3 ± 15.3	34.8 ± 7.4	118.4 ± 24.4	4.2 ± 0.3	6.5 ± 1.5
	Total	69	85.1 ± 17.6^†^	33.3 ± 7.5^†^	115.3 ± 28.8^*^	4.2 ± 0.3^†^	6.7 ± 1.6
Pi_max_ (% pred)	≤80%	14	75.4 ± 20.9	32.8 ± 9.48	117.2 ± 32.3	4.3 ± 0.3	7.5 ± 1.9
	81–100%	15	88.2 ± 16.6	32.1 ± 9.4	120.4 ± 34.0	4.3 ± 0.3	6.8 ± 1.4
	>100	40	87.3 ± 15.9	33.8 ± 5.9	112.7 ± 25.7	4.2 ± 0.3	6.4 ± 1.5
	Total	69	85.1 ± 17.6	33.3 ± 7.5	115.3 ± 28.8	4.2 ± 0.3	6.7 ± 1.6

W_max_, maximal load; VO_2_peak/kg, maximal oxygen consumption/kg; VO_2peak_, expressed relative to BM raised by the exponent of 0.67 (VO_2peak_/kg^0.67^); log-linear adjustment of VO_2peak_ (VO_2peak/kgalo_); Pi_max_, maximal inspiratory pressure; Pe_max_, maximal expiratory pressure.

* p < 0.05.

† *p < 0.01*.

There were no statistically significant differences in age, lung function parameters, exacerbation score or respiratory muscle strength according to gender. Males had better BMI, VO_2peak_/kg, VO_2peak_/kg^−0.67^, VO_2peak−alo_ and ventilatory equivalent for CO_2_ (V_E_/VCO_2_; Table 1).

Simple linear regression showed that Pe_max_, FEV_1_ (%) and RV/TLC (%) were independent statistically significant predictors of W_max_ (Table 3).

**Table 3:** Linear regression with W_max_ as a dependent variable.

**Independent variable**	**Simple linear regression**			**Multiple regression models**		
	***B***	**SE**	***p***	***B***	**SE**	***p***
Pi_max_	11.9	6.1	0.05	−4.5	6.40	0.49
Pe_max_	24.9	5.5	<0.001	20.6	6.75	0.003
FEV_1_ (%)	0.30	0.07	<0.001	0.14	0.22	0.26
RV/TLC (%)	−0.15	0.04	<0.001	−0.04	0.06	0.48

*RV/TLC, residual volume/total lung capacity; Pi_max_, maximal inspiratory pressure; Pe_max_, maximal expiratory pressure*.

**Figure 1:**
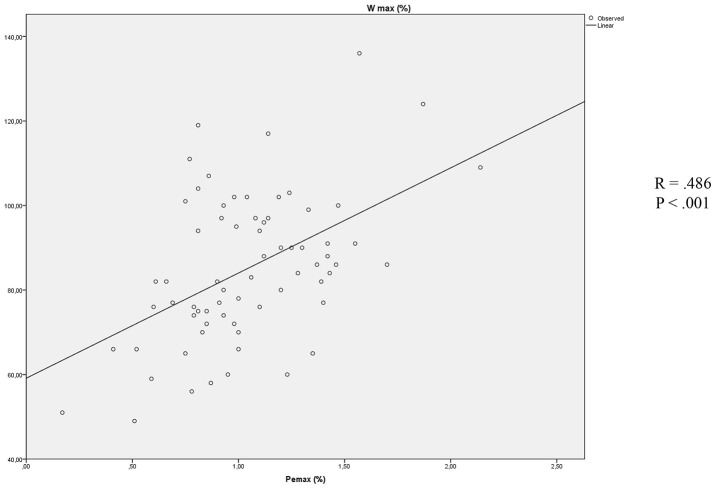
Association between Pe_max_ and W_max_.

Stepwise multiple linear regressions confirmed the value of Pe_max_ (*B* = 24.883, β = 0.483, *p* < 0.001) as the best predictor of W_max_, instead of parameters that were excluded from the analysis (RV/TLC, BMI, Pi_max_, FEV_1_; Figure 1).

## Discussion

Our study showed that CF subjects with decreased expiratory muscles strength had diminished lung function and exercise tolerance. Lower values of lung function and exercise intolerance were not shown to be related to inspiratory muscle strength.

Peripheral muscle dysfunction (both strength and endurance) in patients with CF is probably multifactorial. Divangahi et al. (21) showed in animal models that lack of the cystic fibrosis transmembrane conductance regulator (CFTR) in skeletal myotubules, including the diaphragm, lead to dysregulation of calcium homeostasis, augmentation of inflammatory gene expression, and increased muscle weakness. Although this is an interesting finding, diminished sarcolemmal excitability due to dysfunctional CFTR is only one of several possible causes of reduced muscle strength in patients with CF (22). Probably the most prominent is physical inactivity, which leads to plain myopathy. Myopathy is generally the result of a sedentary lifestyle, and can be improved with physical exercise. Other factors (e.g., systemic inflammation, oxidative stress, frequent exacerbation, use of systemic corticosteroids, malnutrition) also contribute to skeletal muscle atrophy and weakness in CF patients (23).

Although frequent use of systemic corticosteroids in some CF patients may have a negative influence on skeletal muscle strength, the leading factors used to explain the associated respiratory muscle weakness were pulmonary hyperinflation and malnutrition (24–26). Using near-infrared spectroscopy and *P* magnetic resonance spectroscopy with a relatively small group of CF patients displaying preserved lung function, Werkman et al. (27) did not find that intrinsic metabolic abnormalities in oxygenation and muscle oxidative metabolisms contributed to exercise intolerance. Static hyperinflation has also been shown to be an important cause of ventilatory limitation during progressive CPET (28). We have shown a significant negative correlation between Pe_max_ and hyperinflation, which was not the case with Pi_max_. Dassios et al. (7) showed that CF patients with mild lung disease exhibit impaired respiratory muscle function in comparison to healthy controls. Our results, from a smaller group of patients comparable in age, also showed the lowest values of static respiratory pressures in patients with decreased lung function. However, the only difference that proved to be significant was a reduction in expiratory muscle strength. Furthermore, we showed that, during CPET, Pe_max_ was a useful predictor of W_max_, which is a “gold standard” for the estimation of exercise tolerance. In both healthy individuals and in CF patients with mild lung disease, expiratory muscles (mostly abdominal and rib cage muscles) are active only in the case of an increased workload. With this in mind, we think that the primary dysfunction of these muscle groups is caused by rib cage deformity, which leads to secondary muscular inefficacy. Interesting data showing a negative correlation between respiratory pressures and the upper arm muscle area confirmed the importance of complementary estimation of nutritional status as well as BMI calculation (29). In addition, static respiratory pressures correlate with skeletal muscle strength (10). This correlation leads to one of the limitations of our study: it lacked the particular estimation of abdominal and rib cage muscles strength.

We showed a significant positive correlation between FEV_1_ and Pi_max_, a finding that is in accordance with recently publish data on adults with CF (30). However, we found an insignificant correlation between inspiratory muscle strength and exercise capacity during CPET. This finding is in opposition with some previous studies that estimated exercise tolerance using a modified shuttle test (10). Field tests, although being useful in clinical practice are often submaximal and its usefulness may be limited. Leroy (31) indirectly confirmed our results in their research, showing no significant correlation between inspiratory muscle endurance calculated from Pi_max_ and exercise capacity.

Regardless of disease severity, regular physical activity improves exercise tolerance and respiratory muscle strength (32). Exercise programs improve muscle endurance, which influences the sensation of dyspnea during exercise (6, 22). This is of particular importance, knowing that CF patients are at increased risk of respiratory muscle fatigue (29).

Dunnink et al. accentuated decreased respiratory muscle strength in females among CF patients with a mean age of 26 years (10). We did not show such a difference. In fact, females exhibited better mean Pe_max_ and Pi_max_ in our study. This is probably because the lung function results of the younger cohort in our study were not significantly different between males and females, which was the case in the above-mentioned research. Increased VO_2_/kg in males can be explained by gender-dependent skeletal muscle composition.

Normalizing the data using allometric scaling has been proposed as an efficient method when large body mass differences are present (15). We showed that younger participants with milder lung disease had higher values of VO_2peak−alo_ that can be explained by muscle wasting in more severe disease. Nevertheless, age difference was not proved to be significant factor comparing respiratory muscle strength.

We think regular measurement of respiratory muscle strength should be a usual clinical practice for patients with CF, especially because it can be accurately measured with portable mouth pressure manometers. The practice may help in recognizing those patients in need of intensive rehabilitation programs and observed exercise training to improve habitual physical activity and respiratory muscle strength.

## Author contributions

AS contributed for literature search, data collection, study design, analysis of data, manuscript preparation, and review of manuscript. PM contributed for data collection, study design, analysis of data, manuscript preparation, and review of manuscript. GM-S contributed for literature search, study design, analysis of data, manuscript preparation, and review of manuscript. GT contributed for study design, analysis of data, manuscript preparation, and review of manuscript.

### Conflict of interest statement

The authors declare that the research was conducted in the absence of any commercial or financial relationships that could be construed as a potential conflict of interest.
